# Release of Luminal Exosomes Contributes to TLR4-Mediated Epithelial Antimicrobial Defense

**DOI:** 10.1371/journal.ppat.1003261

**Published:** 2013-04-04

**Authors:** Guoku Hu, Ai-Yu Gong, Amanda L. Roth, Bing Q. Huang, Honorine D. Ward, Guan Zhu, Nicholas F. LaRusso, Nancy D. Hanson, Xian-Ming Chen

**Affiliations:** 1 Department of Medical Microbiology and Immunology, Creighton University School of Medicine, Omaha, Nebraska, United States of America; 2 Division of Gastroenterology and Hepatology, Mayo Clinic College of Medicine, Rochester, Minnesota, United States of America; 3 Division of Geographic Medicine and Infectious Diseases, Tufts Medical Center, Boston, Massachusetts, United States of America; 4 Department of Pathobiology, College of Veterinary Medicine, Texas A&M University, College Station, Texas, United States of America; University of Virginia Health System, United States of America

## Abstract

Exosomes are membranous nanovesicles released by most cell types from multi-vesicular endosomes. They are speculated to transfer molecules to neighboring or distant cells and modulate many physiological and pathological procedures. Exosomes released from the gastrointestinal epithelium to the basolateral side have been implicated in antigen presentation. Here, we report that luminal release of exosomes from the biliary and intestinal epithelium is increased following infection by the protozoan parasite *Cryptosporidium parvum*. Release of exosomes involves activation of TLR4/IKK2 signaling through promoting the SNAP23-associated vesicular exocytotic process. Downregulation of *let-7* family miRNAs by activation of TLR4 signaling increases SNAP23 expression, coordinating exosome release in response to *C. parvum* infection. Intriguingly, exosomes carry antimicrobial peptides of epithelial cell origin, including cathelicidin-37 and beta-defensin 2. Activation of TLR4 signaling enhances exosomal shuttle of epithelial antimicrobial peptides. Exposure of *C. parvum* sporozoites to released exosomes decreases their viability and infectivity both *in vitro* and *ex vivo*. Direct binding to the *C. parvum* sporozoite surface is required for the anti-*C. parvum* activity of released exosomes. Biliary epithelial cells also increase exosomal release and display exosome-associated anti-*C. parvum* activity following LPS stimulation. Our data indicate that TLR4 signaling regulates luminal exosome release and shuttling of antimicrobial peptides from the gastrointestinal epithelium, revealing a new arm of mucosal immunity relevant to antimicrobial defense.

## Author Summary

Exosomes are secreted membranous nanovesicles produced by a variety of cells. Exosomes shuttle various molecules to transfer them to neighboring or distant cells, and have been implicated as mediators in cell-cell communications to modulate physiological and pathological procedures. Here, we report that luminal release of exosomal vesicles is an important component of Toll-like receptor 4 (TLR4)-associated gastrointestinal epithelial defense against infection by *Cryptosporidium parvum*, an obligate intracellular protozoan that infects gastrointestinal epithelial cells. Activation of TLR4 signaling in host epithelial cells following *C. parvum* infection promotes luminal release of epithelial exosomes and exosomal shuttling of antimicrobial peptides from the epithelium. By direct binding to the *C. parvum* surface, exosomal vesicles reveal anti-*C. parvum* activity. Activation of TLR4 signaling in epithelial cells after LPS stimulation also increases exosomal release and exosome-associated anti-*C. parvum* activity. Therefore, we speculate that TLR4-mediated exosome release may be relevant to innate mucosal immunity in general, representing a new target for therapeutic intervention for infectious diseases at the mucosal surface.

## Introduction

Eukaryotic cells release membrane vesicles into their extracellular environment under physiological and pathological conditions [1]. These vesicles mediate the secretion of a wide variety of proteins, lipids, mRNAs, and microRNAs (miRNAs), interact with neighboring cells, and thereby traffic molecules from the cytoplasm and membranes of one cell to other cells or extracellular spaces [1], [2]. There is increasing evidence that secreted vesicles play an important role in normal physiological processes, development, and viral infection and other human disease [3]–[6]. Exosomes represent a specific subtype of secreted membrane vesicles that are around 30–100 nm in size, formed inside the secreting cells in endosomal compartments called multi-vesicular bodies (MVBs) [2]. Exosomes are produced by a variety of cells (e.g., reticulocytes, epithelial cells, neurons, tumor cells) and have been found in bronchoalveolar lavage, urine, serum, bile, and breast milk [2], [7], [8]. The composition of exosomes is heterogenic, depending on the cellular origin of the exosome. Exosomes do not contain a random array of intracellular proteins, but a specific set of protein families arising from the plasma membrane, the endocytic pathway, and the cytosol, especially those of endosomal origin, such as CD63, ICAM-1, and MHC molecules [2], [9]–[13].

Secretion of exosomes is regulated by various stimuli, including the activation of P2X receptor by ATP on monocytes and neutrophils, thrombin receptor on platelets, and Toll-like receptor (TLR) 4 by LPS on dendritic cells [2], [14], [15]. Formation of intraluminal vesicles of MVBs and targeting of transmembrane proteins to these vesicles involve a complex intracellular sorting network, including the endosomal sorting complex required for transport (ESCRT) machinery [2], [15]. Fusion of MVBs with plasma membrane is an exocytotic process that requires the association of v-SNAREs (from the vesicles) and t-SNAREs (at the membrane) to form a ternary SNARE (SNAP receptor) complex. The SNARE complex brings the two membranes in apposition, a necessary step in overcoming the energy barrier required for membrane fusion [16]. Several Rab family proteins, including Rab11 and Rab27b, are key regulators of the exosome secretion pathway and are involved in MVB docking at the plasma membrane [17].

Epithelial cells along the mucosal surface provide the front line of defense against luminal pathogen infection in the gastrointestinal tract and are an important component of gastrointestinal mucosal immunity [18], [19]. TLRs recognize discrete pathogen-associated molecular patterns and activate a set of adaptor proteins (e.g., MyD88) and intracellular kinases (e.g., IKKs), leading to the nuclear translocation of transcription factors, such as NF-κB [20]. Activation of the TLR/NF-κB pathway initiates a series of host defense reactions against pathogens, including parasites. Exosomes derived from the apical and basolateral sides of gastrointestinal epithelial cells, including biliary epithelial cells, have recently been identified, but their physiologic and pathologic relevance is still unclear [21], [22]. These basolateral exosomes have been shown to modulate lymphocyte immune responses during mucosal infection [21]. Intestinal epithelial cell-derived exosomes containing αvβ6 integrin and food antigen induced the generation of tolerogenic dendritic cells in a model of tolerance induction [23]. The presence of these intestinal epithelial cell-derived exosomes impacted the development of antigen-specific T regulatory cells [23]. Release of exosomes into the bile has been shown to influence intracellular regulatory mechanisms and modulate biliary epithelial cell proliferation via interactions with epithelial primary cilia [24].

*Cryptosporidium parvum* is an obligate intracellular protozoan of the phylum Apicomplexa that infects intestinal and biliary epithelial cells [25]. Infection activates TLR4 signaling in host epithelial cells through direct parasite-host cell interactions [19], [25]. Due to the “minimally invasive” nature of infection, epithelial cells play a key role in activating and communicating with the host immune system against *C. parvum* infection. MicroRNAs are small regulatory RNAs that mediate either mRNA cleavage or translational suppression, resulting in gene suppression [26]. miRNAs can be seen as a fine-tuning for the cellular responses to external influences, and might be important players in the regulation of host immune response. In our previous studies, we demonstrated that activation of TLR4/NF-κB signaling in epithelial cells regulates transcription of miRNA genes to orchestrate host anti-*C. parvum* immune responses through modulation of miRNA-mediated posttranscriptional suppression [27], [28]. In the work described here, we found that *C. parvum* infection stimulates host epithelial monolayers to release apical exosomes through activation of TLR4 signaling, with the involvement of activation of the IKK2/SNAP23 exocytic sorting and *let-7* miRNA-mediated gene regulation. Released exosomes shuttle several antimicrobial peptides, can bind to the *C. parvum* sporozoite surface, and display anti-*C. parvum* activity *in vitro* and *ex vivo*. Our results demonstrate, for the first time, that luminal release of exosomes is an important component of TLR4-associated epithelial immune reactions against *C. parvum* infection.

## Results

### *C. parvum* Infection Increases Luminal Release of Exosomes from the Host Epithelium through Activating the TLR4/IKK2 Signaling Pathway

**Figure 1:**
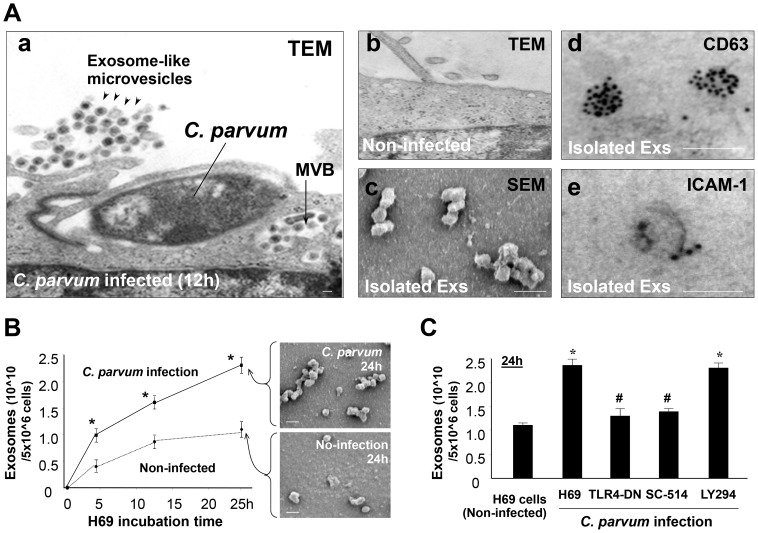
*C. parvum* infection increases luminal release of epithelial exosomes through activating the TLR4 signaling pathway. (A) Infection stimulates luminal release of exosomes (Exs) from H69 monolayers, as assessed by electron microscopy (EM). Abundant exosome-like microvesicles were observed in the apical region of H69 monolayers following infection (arrowheads in a), with multi-vesicular bodies (MVBs) detected in the cytoplasm of infected cells (arrow in a). Only a few microvesicles were detected at the apical region of non-infected monolayers (b). Isolated apical microvesicles are 40–100 nm in size by scanning EM (c) and are positive by immunogold for exosome markers CD63 (d) and ICAM-1 (e). (B) A time-dependent apical exosome release was detected in infected H69 monolayers by nanosight tracking analysis and scanning EM. (C) Inhibition of TLR4 (by TLR4-DN transfection) and IKK2 (by SC-514), but not PI-3K (by LY294002), blocked *C. parvum*-induced exosome release. Scale bars = 100 nm; * p<0.05 ANOVA versus the non-infected controls; ^#^ p<0.05 ANOVA versus infected cells.

Non-malignant human biliary epithelial cells, H69 cells, were grown to confluence on Percoll inserts to form monolayers; we detected a few exosome-like microvesicles in the apical side under electron microscopy (EM) (Figure 1A). These microvesicles were cup-shaped, with a diameter size about 40–100 nm, typical for exosomes. When H69 monolayers were exposed to infection by *C. parvum*, abundant exosome-like microvesicles were found in the apical region by the epithelium (Figure 1A). These microvesicles were morphologically similar to the exosome-like microvesicles found in non-infected monolayers. Interestingly, MVBs were detected in the cytoplasm of infected cells. These MVBs usually contained several exosome-like microvesicles that were morphologically similar to those released to the apical region (Figure 1A). Using a well-established ultracentrifugation approach [29], we isolated and purified these exosome-like microvesicles from the apical supernatants of the H69 monolayers 24h after exposure to *C. parvum* infection. These purified microvesicles displayed cup-shape vesicular characteristics of exosomes, with a diameter size of 40–100 nm under scanning EM. Immunogold staining revealed that these exosome-like microvesicles were positive for exosome markers CD63 and ICAM-1 (Figure 1A), and, thus, they were subsequently referred to as exosomes. Interestingly, these apical exosomes released from H69 monolayers following infection were positive for MHC I and MHC II (Figure S1). We then quantified the release of apical exosomes from H69 monolayers at various time points after exposure to *C. parvum*. A time-dependent increase of apical exosomes was identified using multiple approaches, including Nanoparticle Tracking Analysis (NTA) [30], [31] (Figure 1B and Figure S1), EM (Figure 1B), and Western blot for CD63 (Figure S1).

Having determined the increment of apical exosome release from cell monolayers following *C. parvum* infection, we then tested the potential underlying mechanisms. Previous studies demonstrated that several intracellular signaling pathways are activated in host epithelial cells following infection, including the TLR4/IKK/NF-κB and PI-3K pathways [28], [32]. Transfection of epithelial cells with the TLR4-dominant mutant (TLR4-DN) or treatment of cells with the IKK2 inhibitor SC-514 [33] significantly inhibited *C. parvum*-induced exosome release (Figure 1C). In contrast, an inhibitor of PI-3K showed no effects. Similarly, exposure of H69 monolayers to LPS (a potent TLR4 ligand) increased release of apical exosomes (Figure S1). Release of apical exosomes induced by LPS stimulation or *C. parvum* infection was also noted in cultured mouse biliary epithelial monolayers (603B monolayers) and intestinal epithelial monolayers (IEC4.1 monolayers) (Figure S1).

**Figure 2:**
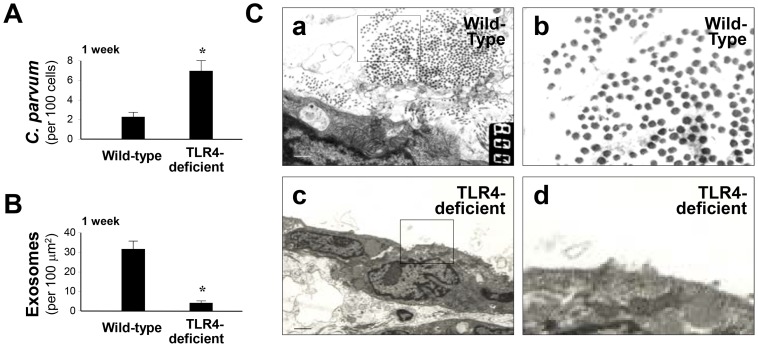
TLR4-dependent luminal release of exosomes during *C. parvum* biliary infection in mice. (A) and (B) Luminal release of biliary exosomes during biliary cryptosporidiosis in the wild type and TLR4-deficient mice. *C. parvum* oocysts were injected into the gallbladder of wild-type or TLR4-deficient mice, and liver tissues were collected one week post-injection. Quantitative analysis detected a higher infection burden and a lower amount of luminal exosome content in TLR4-deficent mice. (C) Abundant exosome-like microvesicles were observed in the luminal region of the wild-type mice one week post-infection, compared with that in the TLR4-deficent mice by transmission EM (b and d are higher magnifications in a and c, respectively). Scale bars = 1 µm; * p<0.05 ANOVA versus the wild-type.

To test *C. parvum*-induced exosome release *in vivo*, we applied a mouse model of biliary cryptosporidiosis through gallbladder injection of *C. parvum* oocysts into wild-type and TLR4-deficient mice [34]. Consistent with our previous results [35], a higher biliary infection burden was found in TLR4-deficient mice compared with the wild-type animals (Figure 2A). Intriguingly, abundant exosomes were detected in the lumen of the biliary tract from the wild-type animals following infection (Figure 2B and 2C). Only a few exosomes were detected in the biliary lumen in the infected TLR4-deficient mice. Taken together, these data suggest that *C. parvum* infection increases luminal release of exosomes from the biliary epithelium, probably through TLR4/IKK2-mediated activation of the MVB exocytotic pathway.

### *C. parvum*-induced Release of Apical Exosomes from Biliary Epithelial Cells Involves Rab- and SNAP23-mediated MVB Exocytosis

**Figure 3:**
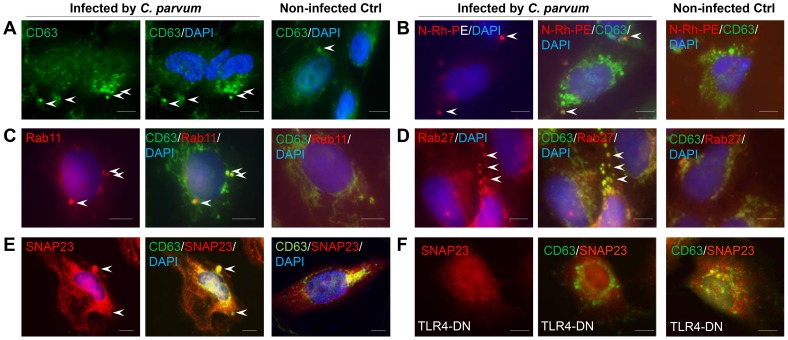
Colocalization of Rab11, Rab27, and SNAP23 to MVBs in epithelial cells following *C. parvum* infection. (A) and (B) Visualization of MVBs by CD63 staining and N-Rh-PE labeling. H69 cells were exposed to *C. parvum* infection for 12 h and stained with CD63 antibody. CD63 staining showed typical patchy intracellular distribution of MVBs in infected cells (arrows in A). Pre-incubation of cells with N-Rh-PE to label MVBs followed by *C. parvum* infection and CD63 staining reveal overlay of CD63 staining with the labeling of newly-formed MVBs (arrows in B). (C) and (D) Co-localization of Rab11 and Rab27b with MVBs in infected cells (indicated by arrows). H69 cells were exposed to *C. parvum* infection for 12 h and co-stained with antibodies to CD63 and Rab11 or Rab27b. (E) and (F) TLR4-dependent co-localization of SNAP23 to MVBs in infected cells. Cells were exposed to *C. parvum* for 12 h and co-stained with antibodies to CD63 and SNAP23. *C. parvum*-induced co-localization of SNAP23 to the MVBs was detected in H69 cells (arrows in E), but not in cells stably expressing TLR4-DN (F). Scale bars = 5 µm.

To test the contribution of MVB-associated exocytosis in *C. parvum*-induced apical exosome release, we performed experiments using confocal analysis on *C. parvum*-infected H69 cells. Using CD63 as the marker for MVBs [2], we detected an increase of MVBs in the cytoplasm of H69 cells following *C. parvum* infection (Figure 3A). The CD63-positive staining showed patchy distribution in the cytoplasm, with a diameter from 300 to 1,000 nm, consistent with characteristics of MVBs in cells as shown by EM in Figure 1A. In addition, these CD63-positive vesicles in the cytoplasm were overlaying with exosomes labeled with N-(Lissamine) rhodamine B sulfonyl dioleoylphosphatidylethanolamine (N-Rh-PE) [36] (Figure 3B), suggesting that they were MVBs. Immunostaining revealed positive reactions to Rab11, Rab27b, and SNAP23 co-localized to the CD63-positive MVBs (Figure 3C–3E). Of note, accumulation of SNAP23 was obviously around the MVBs in infected cells (Figure 3E), which was further confirmed in cells overexpressing SNAP23 (Figure S2). Interestingly, *C. parvum*-induced SNAP23 localization around MVBs was not detected in H69 cells stably expressing TLR4-DN (Figure 3F).

**Figure 4:**
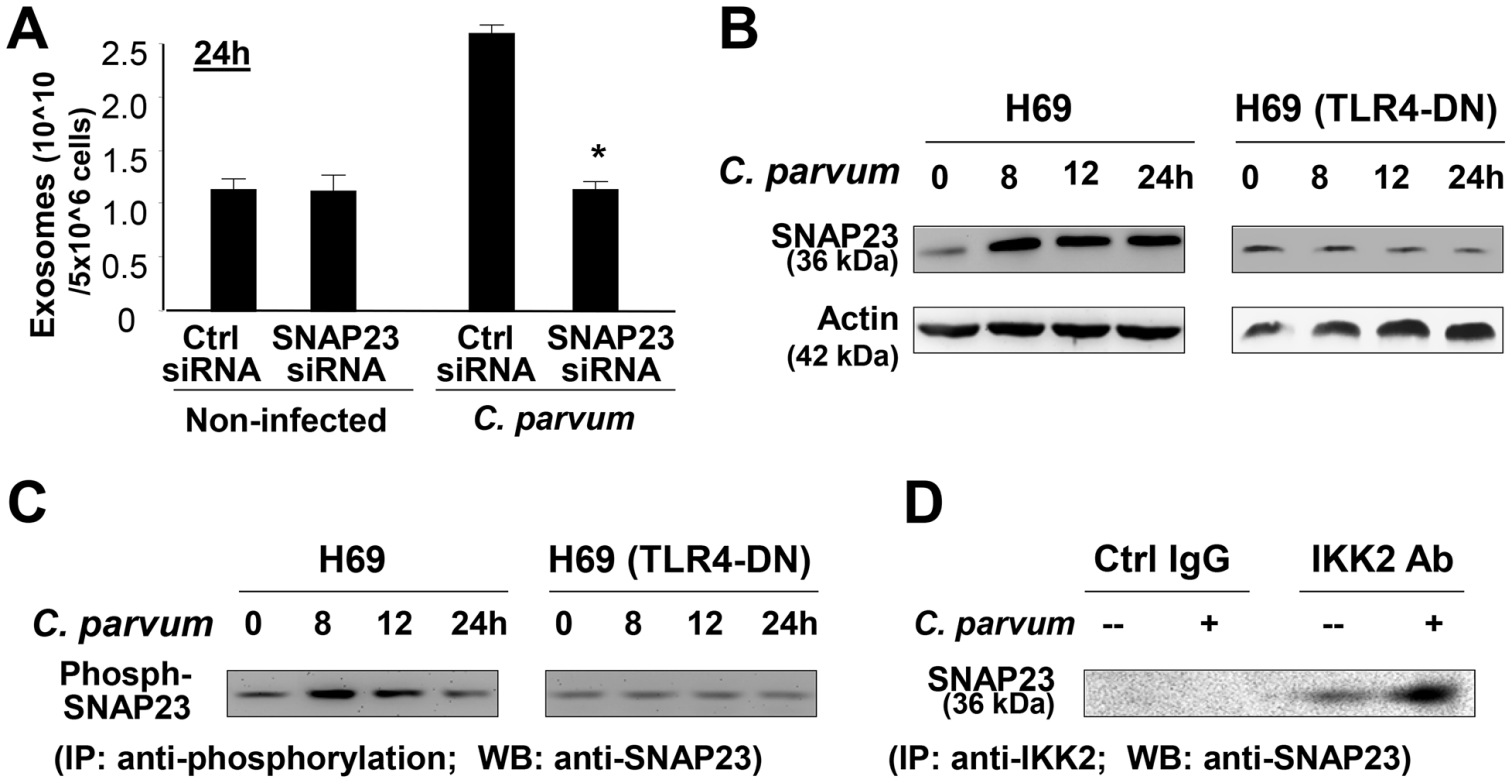
*C. parvum*-induced release of apical exosomes from biliary epithelial cells involves TLR4-dependent IKK2-SNAP23 interactions. (A) SNAP23 siRNA inhibits *C. parvum*-induced release of apical exosomes. H69 monolayers were treated to a non-specific control siRNA or SNAP23 siRNA, followed by exposure to *C. parvum* infection for 24 h. Exosomes released into the apical supernatants were isolated and quantified. (B) and (C) TLR4-dependent induction of SNAP23 expression and its phosphorylation in cells following *C. parvum* infection, as assessed by Western blot (B) and immunoprecipitation (IP) analysis (C). *C. parvum* infection increased total and phosphorylated SNAP23 levels in H69 cells, but not in cells stably expressing TLR4-DN. (D) *C. parvum* infection enhances direct interactions between SNAP23 and IKK2. H69 cells were exposed to *C. parvum* for 24 h, and direct interaction between SNAP23 and IKK2 was measured by IP. * p<0.05 ANOVA versus infected non-specific siRNA control.

To further investigate the role of SNAP23 in *C. parvum*-induced exosome release, H69 cells were first treated with an siRNA to SNAP23, followed by exposure to *C. parvum* infection for 24 h. SNAP23 siRNA treatment resulted in a significant decrease in exosome release from infected H69 monolayers (Figure 4A). H69 cells were infected by *C. parvum* for various time points, followed by measurement of total SNAP23 by Western blot and phosphorylated SNAP23 by immunoprecipitation (IP). Increased levels of total SNAP23 (Figure 4B) and phosphorylated SNAP23 (Figure 4C) were detected in infected cells. *C. parvum*-induced SNAP23 expression and phosphorylation were not detected in H69 cells stably expressing TLR4-DN (Figure 4B). Induction of SNAP23 total protein was detected in mouse biliary epithelial 603B cells infected by *C. parvum* (Figure S3). Upregulation of SNAP23 and enhanced phosphorylation of SNAP23 were also detected in H69 cells following LPS stimulation (Figure S3). Previous studies demonstrated that SNAP23 is a substrate for IKK2, and phosphorylation of SNAP23 at Ser120 and Ser95 by IKK2 stimulates fusion of intracellular vesicles to the cell membrane, promoting exocytosis [37]. Indeed, a direct association between IKK2 and SNAP23 was evident from IP analysis (Figure 4D). Increased interaction between IKK2 and SNAP23 was detected in infected cells (Figure 4D). Such observations, however, do not prove that an IKK2-SNAP23 association is occurring in MVBs or exosomes or that it is just relevant to these processes. Together, these data suggest that TLR4 signaling activates IKK2 to phosphorylate SNAP23 to stimulate exosome release in cells following *C. parvum* infection.

### miRNAs of the *let-7* Family Target SNAP23, and Downregulation of *let-7* miRNAs Is Associated with *C. parvum*-induced Exosome Release from Epithelial Cells

**Figure 5:**
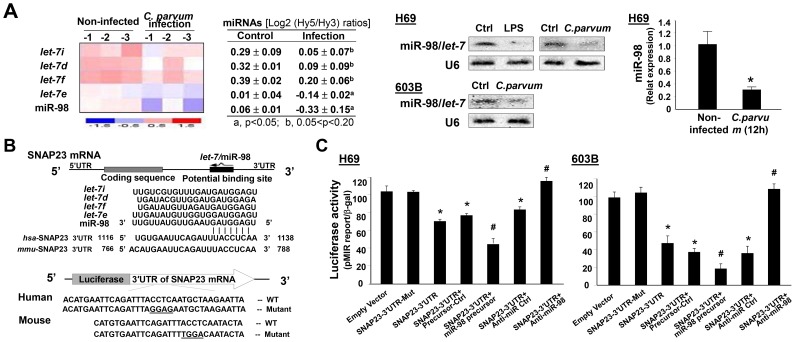
The *let-7* miRNAs target SNAP23 and are downregulated in epithelial cells following *C. parvum* infection. (A) *C. parvum* infection downregulates expression of the *let-7* miRNA family. H69 cells were exposed to *C. parvum* infection for 12 h, followed by miRNA analysis using mercury LNA array, Northern blot, and real-time PCR. A heat map of the *let-7* miRNA family is shown and expression levels are presented as the log_2_ (Hy^5^/Hy^3^) ratios. Representative Northern blot for *let-7*/miR-98 in H69 and 603B cells and real-time PCR quantification of miR-98 in H69 cells are shown. (B) The schematic of SNAP23 mRNA showed a potential binding site in its 3′UTR for the *let-7* miRNA family in humans and in mice. The SNAP23 3′UTR sequence covering the potential binding sites for the *let-7* miRNA family was inserted into the pMIR-REPORT luciferase plasmid. A control plasmid with the mutant 3′UTR sequence was also generated for control. (C) Binding of *let-7*/miR-98 miRNAs to the potential binding site in the SNAP23 3′UTR results in translational suppression. Cells were transfected with the pMIR-REPORTER luciferase constructs and treated with anti-miRs or precursors to miR-98, or non-specific oligo control, for 24 h, followed by luciferase analysis. *, p<0.05 ANOVA versus the non-infected control (in A) or empty vector control (in C); ^#^, p<0.05 ANOVA versus the control precursor or control anti-miR (in C).

miRNAs, such as the *let-7* family members, have been implicated in the regulated exocytotic process [38]–[40]. Interestingly, several members of the *let-7* miRNAs, including *let-7i*, *let-7d*, *let-7f*, *let-7e*, and miR-98, showed significant downregulation in H69 cells following *C. parvum* infection by array analysis (Figure 5A), consistent with our previous studies on H69 cells [27], [28]. Decreased expression of *let-7i* and miR-98 was further confirmed by Northern blot and real-time PCR analysis in H69 and 603B cells following *C. parvum* infection (Figure 5A). Consistent with results from our previous studies on LPS-suppressed expression of *let-7* miRNAs in epithelial cells [41], we detected a decreased expression level of miR-98 in H69 cells following LPS stimulation (Figure S3). Using *in silico* database analysis, we found that there is conserved complementarity between SNAP23 3′UTR and miRNAs of the *let-7* family (Figure 5A). To test the potential targeting of SNAP23 mRNA by *let-7* miRNAs, we generated pMIR-REPORT luciferase constructs containing the SNAP23 3′UTR with the putative *let-7* binding site (Figure 5B). In addition, constructs with the CCTC to GGAG for human and ACCT to TGGA for mouse mutation at the putative binding sites were also generated as controls. We then transfected H69 cells with these reporter constructs, followed by assessment of luciferase activity 24 h after transfection. As shown in Figure 5C, a significant decrease of luciferase activity was detected in cells transfected with the SNAP23 3′UTR construct containing the potential binding site compared with mutant control vector. No change in luciferase activity was observed in cells transfected with the mutant SNAP23 3′UTR construct, suggesting endogenous translational repression of the construct with the SNAP23 3′UTR. Anti-miRs (anti-miR miRNA inhibitors) and miRNA precursors are chemically modified RNA molecules designed to specifically inhibit and mimic, respectively, endogenous miRNAs [42]. Accordingly, anti-*let-7i* markedly increased SNAP23 3′UTR-associated luciferase reporter translation (Figure 5C). In contrast, the miR-98 precursor significantly decreased the luciferase activity (Figure 5C). Of note, anti-*let-7i* and miR-98 precursor only caused a modest alteration in the luciferase activity, probably due to the targeting of SNAP23 3′UTR by other members of the *let-7* miRNA family in the cells. Similar results were also confirmed in 603B cells (Figure 5C).

**Figure 6:**
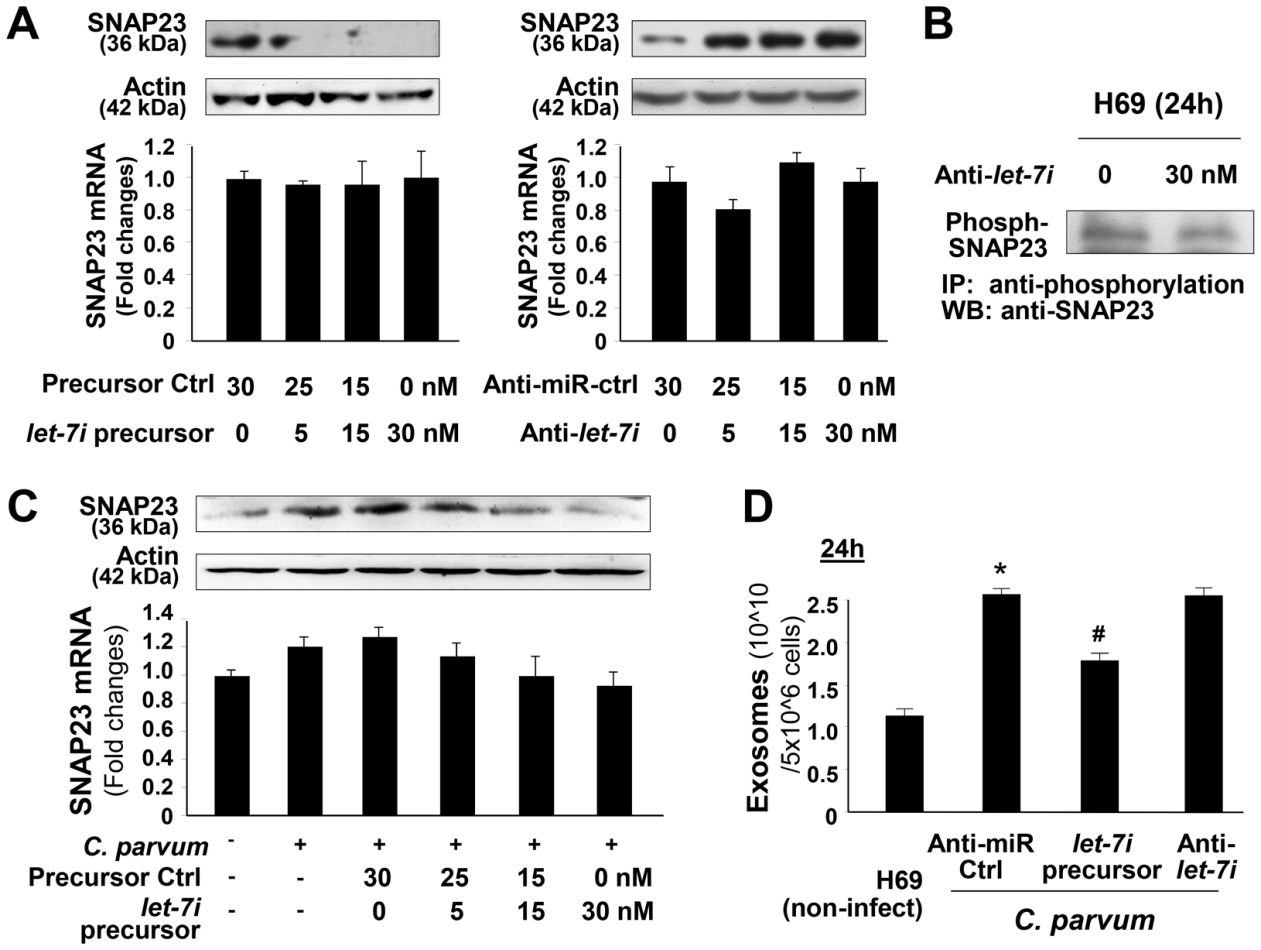
Downregulation of *let-7* miRNAs is associated with *C. parvum*-induced exosome release from epithelial cells. (A) Functional manipulation of *let-7*/miR-98 causes reciprocal alterations in SNAP23 expression at the mRNA and protein levels. H69 cells were treated with various doses of *let-7i* precursor or anti-*let-7i* for 24 h, followed by Western blot for SNAP23 protein (upper panel) and real-time PCR of SNAP23 mRNA (lower panel). Whereas a significant decrease or increase in SNAP23 protein content was detected in cells treated with *let-7i* precursor or anti-*let-7i*, respectively, no changes in SNAP23 mRNA levels were observed in the treated cells. (B) Anti-*let-7i* shows no effect on the phosphorylation of SNAP23 in H69 cells. Cells were treated with anti-*let-7i* for 24 h, followed by IP for phosphorylated SNAP23. (C) Overexpression of *let-7i* blocks *C. parvum*-induced upregulation of SNAP23. H69 cells were transfected with *let-7i* precursor for 48 h and then exposed to *C. parvum* infection for 24 h. Expression of SNAP23 was measured by real-time PCR and Western blot. (D) Functional manipulation of *let-7i* causes alterations in *C. parvum*-induced release of apical exosomes. H69 monolayers were treated with a non-specific anti-miR control or anti-*let-7i* or *let-7i* precursor, followed by exposure to *C. parvum* infection for 24 h. Exosomes released into the apical supernatants were isolated and quantified. Data are averages of three independent experiments. *, p<0.05 ANOVA versus the non-infected control; ^#^, p<0.05 ANOVA versus the control anti-miR.

To test whether miRNA-mediated suppression of SNAP23 is directly relevant to SNAP23 expression in biliary epithelial cells, we treated H69 cells with miR-98 precursor or anti-*let-7i* and then measured SNAP23 protein level (treated for 48 h) by Western blot or mRNA level (treated for 24 h) by real-time PCR. Treatment of cells with the anti-*let-7i* caused a significant increase in SNAP23 protein content (Figure 6A). Conversely, a decrease in SNAP23 protein level was detected in cells after treatment with the miR-98 precursor (Figure 6A). No significant changes in SNAP23 mRNA level were detected in cells after treatment with miR-98 precursor or anti-*let-7i* (Figure 6A). No significant changes in the phosphorylated SNAP23 level were detected in cells after treatment with the anti-*let-7i* (Figure 6B). These data suggest that *let-7* family miRNAs target SNAP23 3′UTR, resulting in posttranscriptional suppression through translation suppression.

To test the impact of *let-7* miRNAs on *C. parvum*-induced SNAP23 upregulation in H69 cells, we treated cells with the miR-98 precursor for 48 h and then exposed them to *C. parvum* for 24 h, followed by Western blot for SNAP23. The miR-98 precursor diminished *C. parvum*-induced SNAP23 expression (Figure 6C). Moreover, the miR-98 precursor partially blocked *C. parvum*-induced exosome release in H69 cells (Figure 6D). Interestingly, treatment of cells with the anti-*let-7i* showed no significant effects on *C. parvum*-induced exosome release (Figure 6D), presumably because of the low level of *let-7i* in the infected cells as shown in Figure 5A.

### Released Apical Exosomes Display Anti-*C. parvum* Activity through Binding to the *C. parvum* Surface

**Figure 7:**
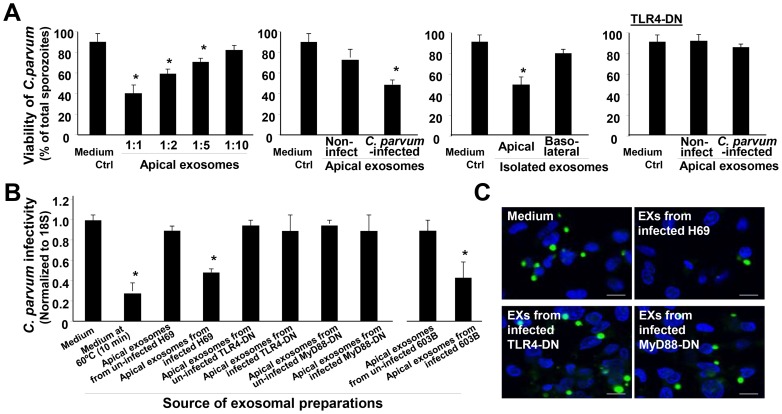
Apical exosomes released from *C. parvum*-infected biliary epithelium display anti-*C. parvum* activity. (A) Effects of incubation with isolated exosomes from the biliary epithelium on *C. parvum* viability. Exosomes were isolated from the basolateral or apical supernatants of H69 monolayers of non-infected control, cells of *C. parvum* infection for 24 h, and cells stably expressing TLR4-DN after infection for 24 h. Freshly excysted *C. parvum* sporozoites were incubated with isolated exosomes (as indicated) for 2 h, and parasite viability was assessed by propidium iodide staining. (B) and (C) Effects of incubation with isolated apical exosomes from the biliary epithelium on the infectivity of *C. parvum* sporozoites. The same number of freshly excysted *C. parvum* sporozoites was incubated with exosomes isolated from H69 or 603B monolayers for 2 h and then added to H69 cells for 2 h. Parasite infection burden was measured by real-time PCR (B) and fluorescence microscopy (C). Pre-incubation of isolated exosomes abolished their inhibitory effects on *C. parvum* infectivity. *C. parvum* parasites were stained in green using a specific antibody and cell nuclei in blue by DAPI. Scale bars = 5 µm; *, p<0.05 ANOVA versus medium control.

To investigate the potential effects of released apical exosomes on *C. parvum*, we incubated freshly excysted *C. parvum* sporozoites with a serial dilution of exosomes isolated from H69 monolayers after exposure to *C. parvum* for 24 h. Incubation of *C. parvum* sporozoites with isolated exosomes resulted in a decrease in *C. parvum* viability in a dose-dependent manner by the viability assay (Figure 7A). Sporozoites incubated with the exosomes isolated from non-infected monolayers showed a modest decrease in *C. parvum* viability (Figure 7A). Exosomes isolated from the basolateral side of infected H69 monolayers, or apical exosomes from infected H69 cells expressing TLR4-DN, showed no significant effects on *C. parvum* viability (Figure 7A). A decrease in *C. parvum* viability was also detected after incubation with apical exosomes isolated from H69 monolayer following LPS stimulation (Figure S3). We then incubated the same number of *C. parvum* sporozoites with apical exosomes isolated from infected H69 monolayers for 2 h; after extensive washing, these sporozoites were added to H69 cells for infection. A decreased infectivity to host cells for *C. parvum* sporozoites after pre-incubation with exosomes was detected, compared with the sporozoites after incubation with culture medium, as assessed by real-time PCR (Figure 7B) and immunostaining (Figure 7C). Incubation with exosomes from the apical side of non-infected H69 monolayers, or infected cells stably expressing TLR4-DN and MyD88-DN, showed no inhibitory effects on *C. parvum* sporozoite infectivity (Figure 7B and 7C). A decreased infectivity of *C. parvum* sporozoites to intestinal IEC4.1 cells after pre-incubation with exosomes was also detected by real-time PCR (Figure S4).

**Figure 8:**
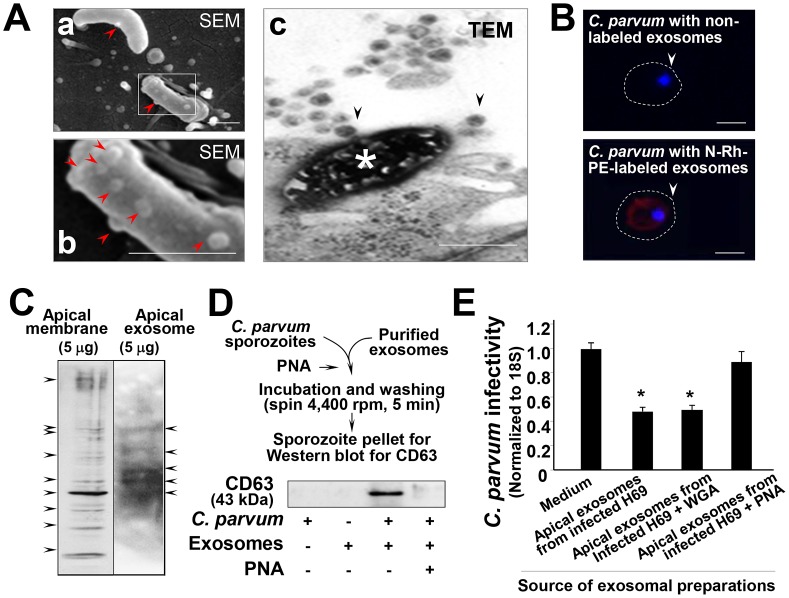
Gal/GalNAc-associated molecules mediate direct binding of exosomes to the *C. parvum* surface. (A) Direct binding of apical exosomes to the *C. parvum* surface observed under EM. When sporozoites were added to H69 cells for 2 h, exosome-like microvesicles on their surface were observed, as evident by SEM (arrows in a and b; b shows a higher magnification of the boxed region in a). The TEM image shows a dying *C. parvum* parasite that was invading an epithelial cell with several exosomes binding to its surface (arrows in c). (B) Delivery of exosomal content to *C. parvum* sporozoites after incubation. Isolated exosomal vesicles were labeled with the N-Rh-PE dye, and after extensive washing, were incubated with *C. parvum* sporozoites for 2 h. These sporozoites showed positive labeling of the dye after incubation. Parasite nuclei were labeled in blue with DAPI (arrows). (C) Gal/GalNAc-associated molecules in H69 apical membrane fraction and exosomes released from H69 monolayers. Positive bands were detected in both H69 apical membrane fraction and released exosomes using SDS gel lectin-blotting with the Gal/GalNAc-specific lectin PNA. (D) Blotting of whole lysates of *C. parvum* sporozoites after pre-incubation with isolated exosomes revealed a positive reaction to exosome marker CD63. (E) PNA attenuates the inhibitory effects of exosomes on *C. parvum* viability. Pre-incubation of isolated exosomes abolished their inhibitory effects on *C. parvum* viability. Scale bars = 1 µm; *, p<0.05 ANOVA versus medium control.

Notably, binding of exosomes to the *C. parvum* sporozoite surface after incubation with apical exosomes was detected by scanning EM. In H69 cell cultures after exposure to *C. parvum* for 2 h, we observed binding of exosomes to the parasite surface (Figure 8A). Some of the sporozoites with several exosomes binding to their surface were dying (showing high electrical density, as indicated by the asterisk in Figure 8A). To further confirm the direct binding of exosomes to the *C. parvum* sporozoite surface, we first labeled exosomes with the N-Rh-PE as previously reported [36]. Labeled exosomes were then incubated with freshly excysted *C. parvum* sporozoites, followed by observation under confocal microscopy. Fluorescent activity was detected in these sporozoites incubated with the labeled exosomes (Figure 8B). No detectable fluorescent activity was observed in the sporozoites incubated with the non-labeled exosomes.

Direct interactions between the Gal/GalNAc-containing glycoproteins on the epithelial cell apical membrane surface and Gal/GalNAc-ligand molecules on the *C. parvum* sporozoite surface have been implicated to mediate the attachment of *C. parvum* sporozoites to host cells [25]. The Gal/GalNAC-specific lectin PNA can markedly decrease the infection [43], [44]. We found that isolated apical exosomes carry Gal/GalNAc molecules (recognized by PNA), displaying positive reactions to PNA blotting at the epithelial apical membrane (Figure 8C). Interestingly, blotting of whole lysates of *C. parvum* sporozoites after pre-incubation with isolated exosomes revealed a positive reaction to exosome marker CD63 (Figure 8D). Moreover, pre-incubation of isolated exosomes with PNA (followed by extensive washing and ultracentrifugation to clear out unbound/free PNA) abolished the inhibitory effects of exosomes on *C. parvum* viability (Figure 8E). The above data indicate that apical exosomes released from *C. parvum*-infected biliary epithelium possess anti-*C. parvum* capacity. Interestingly, exosomes isolated from *C. parvum*-infected H69 monolayers showed no detectable inhibitory effects on the viability of the K12 strain *E. coli* after incubation *in vitro* (Figure S5).

### Apical Exosomes Released from *C. parvum*-infected Biliary Epithelium Carry Antimicrobial Peptides, a Process that Is Mediated by TLR4 Signaling

**Figure 9:**
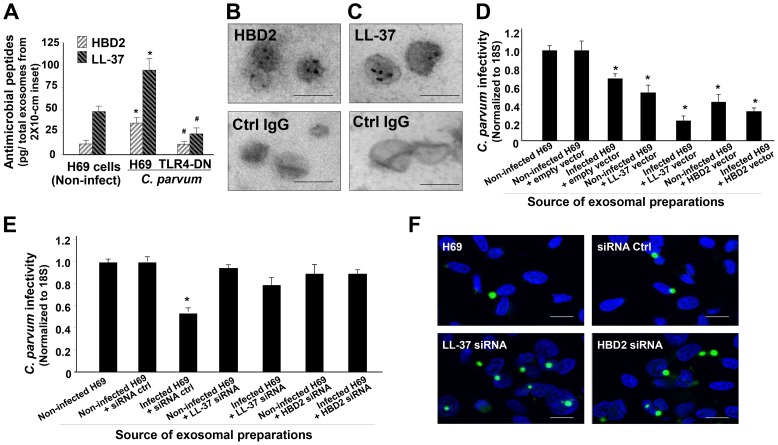
Apical exosomes released from infected cells carry antimicrobial peptides, a process mediated by TLR4 signaling. (A) Apical exosomes released from *C. parvum*-infected H69 monolayers carry antimicrobial peptides in a TLR4-dependent manner. Monolayers of H69 cells and cells stably expressing TLR4-DN were exposed to *C. parvum* for 24 h, and apical exosomes were collected and purified. The content of beta-defensin 2 (HBD2) and cathelicidin 37 (LL-37) in these isolated exosomes was assessed by ELISA. (B) and (C) Existence of HBD2 (B) and LL-37 (C) in apical exosomes from infected H69 monolayers as confirmed by immunogold. Non-specific anti-IgG was used as the control for immunogold. (D)–(F) Functional manipulation of HBD2 and LL-37 expression in H69 cells influences the anti-*C. parvum* activity of their released exosomes by *C. parvum* infection. H69 cells were transfected with the full lengths of HBD2 or LL-37, or siRNAs to HBD2 or LL-37. Cells were then exposed to *C. parvum* infection for 24 h and apical exosomes were isolated. These vesicles were then incubated with freshly excysted *C. parvum* sporozoites for 2 h, and after extensive washing, the sporozoites were added to H69 cells for infection. Parasite infection burden was measured by real-time PCR (D and E) and fluorescent microscopy (F). *C. parvum* parasites were stained in green and cell nuclei in blue. Scale bars = 100 nm (in B and C) and 5 µm (in F); *, p<0.05 ANOVA versus the non-infected control; ^#^, p<0.05 ANOVA versus the infected H69 cells.

Production of antimicrobial peptides, such as beta-defensins and cathelicidins, is a major element for TLR-mediated epithelial anti-*C. parvum* defense [19], [25], [45]. Increasing amounts of human beta-defensin 2 (HBD2) and cathelicidin-37 (LL-37) were detected by ELISA in apical exosomes isolated from *C. parvum*-infected H69 monolayers, compared with those isolated from non-infected cells (Figure 9A). In contrast, no increase in HBD2 and LL-37 contents was detected in the isolated exosomes from infected H69 cells stably expressing TLR4-DN (Figure 9A). Exosomal shuttling of HBD2 and LL-37 was further confirmed by immunogold staining, using the apical exosomes isolated from *C. parvum*-infected H69 monolayers (Figure 9B and 9C). Unfortunately, we have been unable to achieve efficient exosome preparations to demonstrate that the antimicrobial peptides in the exosomes are biochemically active. Therefore, to elucidate whether exosomal shuttle of HBD2 and LL-37 accounts for the anti-*C. parvum* activity of these isolated exosomes from *C. parvum*-infected cells, we performed the gain- and loss-of-function experiments through overexpression or knockdown of HBD2 and LL-37 in H69 cells. These cells were then infected with *C. parvum* for 24 h, and the released exosomes were isolated and then incubated with freshly excysted *C. parvum* sporozoites. Of note, pre-incubation of sporozoites with the exosomes isolated from cells overexpressing HBD2 and LL-37 resulted in a significant decrease in parasite infectivity (Figure 9D). This is probably due to an increased sorting of antimicrobial peptides into the exosomes in these cells at the basal condition. Moreover, after incubation with exosomes isolated from *C. parvum*-infected H69 cells overexpressing HBD2 and LL-37, *C. parvum* sporozoites showed a further decrease in infectivity (Figure 9D). In contrast, incubation with exosomes isolated from *C. parvum*-infected H69 cells with knockdown of HBD2 and LL-37 showed no significant effects on the infectivity of *C. parvum* sporozoites (Figure 9E and 9F). These data suggest that antimicrobial peptides HBD2 and LL-37 in apical exosomes that are released from epithelial cells contribute to mucosal immune defense in response to *C. parvum* infection.

## Discussion

One of the major findings of this study is that activation of TLR4 signaling increases luminal release of exosomes from the biliary epithelium during *C. parvum* infection. Whereas a basal level of exosomal luminal release exists in cultured biliary epithelial monolayers and in the murine biliary tract, a TLR4-dependent increase in luminal release of epithelial exosomes was detected following *C. parvum* infection. Release of exosomes to the extracellular environment involves fusion of MVBs with the plasma membrane. Intracellular trafficking and fusion of compartments classically require small GTPases of the Rab family [2]. Further supporting this concept and consistent with results from previous reports on induced exosome release [17], we detected a significant co-localization of Rab11 and Rab27b with MVBs in the cytoplasm of epithelial cells following *C. parvum* infection. Besides Rab11 and Rab27b, we identified that SNAP23 may regulate the fusion of MVBs with the plasma membrane required for exosome release. *C. parvum* infection increases the protein content of total SNAP23 and enhances phosphorylation of SNAP23 in infected cells. Intriguingly, activation of TLR4 may contribute to both events: TLR4 signaling increases SNAP23 protein expression through modulation of *let-7*-mediated gene regulation, and stimulates SNAP23 phosphorylation through activation of IKK2. We previously demonstrated TLR4/NF-κB-dependent downregulation of *let-7* family miRNAs in *C. parvum*-infected biliary epithelial cells [27], [41]. Targeting of the 3′UTR of SNAP23 by *let-7* family miRNAs resulted in translational suppression. Functional manipulation of *let-7i* caused reciprocal alterations in cellular SNAP23 protein content. Indeed, overexpression of *let-7* miRNA members attenuated *C. parvum*-induced SNAP23 expression and exosome release from the biliary epithelium. In addition, *C. parvum*-induced phosphorylation of SNAP23 is dependent on TLR4 signaling. Knockdown of TLR4 blocked *C. parvum*-induced phosphorylation of SNAP23 in infected cells and, consequently, exosome release into the supernatants. Our data were supported by a recent report showing that SNAP23 is a substrate for IKK2 [37]. Phosphorylation of SNAP23 at Ser120 and Ser95 by IKK2 stimulates fusion of intracellular vesicles to the cell membrane, promoting exocytosis [37]. The next challenge will be to examine in greater depth the involvement of TLR4 signaling in *C. parvum*-induced co-localization of Rab11 and Rab27b with MVBs, and to determine the magnitudes of miRNA-mediated SNAP23 expression versus its phosphorylation in TLR4-regulated exosome release.

Another key point from this study is that released exosomes shuttle antimicrobial peptides and display anti-*C. parvum* capacity, thus contributing to mucosal anti-*C. parvum* defense. It has been reported that antimicrobial peptides, such as HBD2 and LL-37, are present in breast milk and provide protection for maternal breast tissue and the developing digestive tracts of newborns [46]. Additionally, antimicrobial peptides were identified in cervical-vaginal fluid, suggesting their involvement in extracellular immunology [47]. Notably, exosomes have been reported in breast milk and cervical-vaginal fluid [1], [2]. The anti-*C. parvum* activity of HBD2 was previously reported in intestinal and biliary cryptosporidiosis [19], [45]. Here, we identified shuttling of both HBD2 and LL-37 in the apical exosomes released from H69 monolayers. An increase in HBD2 and LL-37 content was detected in the apical exosomes from infected cells, and inhibition of TLR4 signaling decreased their exosomal content. Because these exosomes display a protein profile different from the whole cell lysate, targeting of cytoplasmic proteins to the MVBs within epithelial cells, such as HBD2 and LL-37, must be controlled through highly selective and regulated mechanisms. However, how TLR4 signaling regulates targeting of antimicrobial peptides to MVBs in epithelial cells remains largely unknown. One additional unanswered question is whether shuttling of MHC proteins in the apical exosomes contributes to antigen presentation.

Anti-*C. parvum* activity of apical exosomes released from the epithelium may involve direct binding to the *C. parvum* sporozoite surface. Morphologically, direct binding of exosomes to the *C. parvum* sporozoite surface was evident by scanning and transmission EM observations. Confocal analysis with exosomal labeling confirmed the delivery of exosomal content to *C. parvum* sporozoites after incubation. It is not clear what mechanisms are responsible for this exosome binding/targeting. The infection process of epithelial cells by *C. parvum* is initiated by the attachment of the parasite to the plasma membrane of host epithelial cells [25]. Unidentified specific molecules on the surface of both epithelial cells and *C. parvum* sporozoites mediate this attachment process [43]. Gal/GalNAc epitopes of glycoproteins on the epithelial apical membrane and Gal/GalNAc-specific sporozoite surface lectins are involved in the mechanism(s) of *C. parvum* attachment to intestinal and biliary epithelial cells [43], [44]. As expected, treatment of exosomes with Gal/GalNAc-specific PNA lectin diminished their anti-*C. parvum* activity, suggesting that these molecules may be involved, at least partially, in exosomal binding to the *C. parvum* sporozoite surface. The lifecycle of *C. parvum*, both *in vitro* and *in vivo*, has extracellular stages (i.e., sporozoites, merozoites, and microgametocytes) [25], and they are vulnerable to exosomal binding/targeting. Therefore, luminal release of exosomes from epithelial cells may represent an important element of TLR-mediated mucosal anti-*C. parvum* defense. Surprisingly, it appears that released exosomes have no significant effect on the viability of the K12 strain *E. coli*. One possibility is that the membrane structure of *E. coli* may not favor direct exosomal binding.

In addition, increased exosomal release and exosome-associated anti-*C. parvum* activity were detected in biliary epithelial cells after LPS stimulation. Therefore, TLR4-mediated exosome release may be relevant to innate mucosal immunity in general. Overall, our data suggest that activation of TLR4 signaling stimulates the biogenesis and luminal release of antimicrobial peptide-shuttling exosomes and contributes to gastrointestinal mucosal anti-*C. parvum* defense. Such a process may be explored for therapeutic intervention. Luminal release of exosomes may also mediate the function of distant cells along the gastrointestinal tract, or regulate the homeostasis of gut microbiota, through delivery signaling molecules.

## Materials and Methods

### Ethics Statement

This study was carried out in strict accordance with the recommendations in the Guide for the Care and Use of Laboratory Animals of the National Institutes of Health under the Assurance of Compliance Number A3348-01. All animal experiments were done in accordance with procedures (protocol number # 0868) approved by the Institutional Animal Care and Use Committee of the Creighton University School of Medicine. All surgery was performed under ketamine and xylazine anesthesia, and all efforts were made to minimize suffering.

### *C. parvum* and Cell Lines

*C. parvum* oocysts of the Iowa strain were purchased from a commercial source (Bunch Grass Farm, Deary, ID). Before infecting cells, oocysts were excysted to release infective sporozoites as previously described [19]. H69 cells (a gift from Dr. D. Jefferson, Tufts University,) are SV40-transformed normal human biliary epithelial cells originally derived from normal liver harvested for transplant [19], [27]. 603B cells are immortalized normal mouse biliary epithelial cells (a gift from Dr. Y. Ueno, Tohoku University School of Medicine, Japan). Murine intestinal epithelial cell line (IEC4.1) was a kind gift from Dr. Pingchang Yang (McMaster University, Hamilton, Canada).

### Plasmids and Reagents

TLR4-DN mutant was kindly provided by Dr. M. F. Smith (University of Virginia) and MyD88-DN was a gift from Prof. J. Tschopp (University of Lausanne, Switzerland). Cells stably expressing TLR4-DN or MyD88-DN were established as previously reported [19]. Primers used to amplify the open reading frame of human SNAP23 (NM_130798) were: 5′-GTCATGGATAATCTGTCATCAGAAGAAAT-3′ (forward) and 5′-TTAGCTGTCAATGAGTTTCTTTGCTC-3′ (reverse). PCR products were cloned into pcDNA3.3-TOPO vector (Invitrogen). Primers used to amplify the open reading frame of human beta-defensin 2 (NM_004942) and cathelicidin-37 (NM_004345) were: 5′-CAAGCTTCGATGAGGGTCTTGTATCTCCTCTTCTC-3′ (beta-defensin 2 forward) and 5′-CGGATCCTGGCTTTTTGCAGCATTTTG-3′ (beta-defensin 2 reverse) and 5′-CAAGCTTCGGCTATGGGGACCATGAAGACCC-3′ (cathelicidin-37 forward) and 5′-CGGATCCGGACTCTGTCCTGGGTACAAGATTC-3′ (cathelicidin-37 reverse). PCR products were cloned into the *Hin*dIII and *Bam*H I sites of the pEGFPN3 vector (Invitrogen). WGA and PNA lectins were purchased from Sigma.

### Infection Models and Infection Assays

Models of human biliary cryptosporidiosis using H69 and 603B cells were employed as previously described [19], [27], [28], [41], [43]. Infection was done in a culture medium consisting of DMEM-F12, 100 U/ml penicillin, 100 µg/ml streptomycin, and freshly excysted sporozoites (from oocysts in a 3∶1 ratio with host cells). All excysted sporzoite preparations were tested to exclude LPS contamination using the Limulus Amebocyte Lysate (LAL) gel formation test [48]. Inactivated organisms (treated at 65°C for 30 min) were used for the non-infected controls and experiments were performed in triplicate.

For *in vivo* biliary infection, C57BL/6 wild-type mice and TLR4-deficient (C57BL/10ScNJ; Tlr4^lps-del^/Tlr4^lps-del^ genotype) mice were obtained from Jackson Laboratories. *C. parvum* oocysts were directly injected into the gallbladder of C57BL/6J or TLR4-deficient mice, as previously reported [32], [33]. *C. parvum* infection and exosome release in the intrahepatic bile ducts were observed one week post-injection. Five animals from each group were sacrificed and livers were obtained for immunohistochemistry and electron microscopy.

Real-time PCR, immunofluorescence microscopy, and immunohistochemistry were used to assay *C. parvum* infection as previously reported [17], [27], [28], [41], [43]. Primers specific for *C. parvum* 18s ribosomal RNA (forward: 5′-TAGAGATTGGAGGTTGTTCCT-3′, and reverse: 5′-CTCCACCAACTAAGAACGGCC-3′) were used to amplify the cDNA specific to the parasite. For immunofluorescence microscopy, cells were fixed with 2% paraformaldehyde and incubated with a polyclonal antibody against *C. parvum* [19], followed by anti-rabbit FITC-conjugated secondary antibody and co-stained with 4,6-diamidino-2-phenylindole (DAPI) (Molecular Probes). The liver tissues were stained with H&E, and parasite burden in the biliary tree was performed by counting all intracellular parasite stages in identified bile ducts, as previously reported [35].

### Exosome Purification and Nanoparticle Tracking Analysis (NTA)

Supernatant medium from the apical reservoir of the Percoll inserts was collected at the indicated time points after infection. Medium was then harvested and centrifuged at 1000 rpm for 10 min to eliminate cells and again spun at 10,000 rpm for 30 min, followed by filtration through 0.22 µm filter to remove cell debris. Exosomes were pelleted by ultracentrifugation (Beckman Ti70 rotor) at 44,000 rpm for 70 min and precipitated using exosome precipitation solution (Exo-Quick; System Bioscience) following the manufacturer's instructions. Exosomes were further quantified by the NTA analysis using a Nanosight (model NS500), as previously reported [30], [31].

### Immunofluorescence and Electron Microscopy (EM)

Cell cultures were fixed with 4% paraformaldehyde and incubated with antibodies (Santa Cruz) to SNAP23, CD63, Rab11, and Rab27, followed by treatment with Alexa Fluor 594–conjugated anti-rabbit secondary antibody (1∶100; Invitrogen). Cell cultures were then mounted in Slow Fade anti-fade reagent with DAPI (Molecular Probes). Images were captured using an inverted fluorescence microscope TE2000-E (Nikon).

EM was performed as previously described [24]. Briefly, for transmission EM, exosome pellets were resuspended in 2.5% glutaraldehyde, embedded with a mixture of 4% uranyl acetate and 2% methylcellulose (1∶9 ratio), and observed with a JEOL 1400 electron microscope (JEOL USA). For scanning EM, isolated exosomes were fixed immediately in 2.5% glutaraldehyde, dehydrated, dried in a critical point drying device, sputter coated, and examined with a Hitachi S-4700 microscope (Hitachi). For immunogold analysis, exosomes were fixed in 4% paraformaldehyde, blocked with 10% FCS-PBS for 20 min, and incubated overnight at 4°C with antibodies to CD63 (Santa Cruz), ICAM-1 (Santa Cruz), LL-37 (Hycult Biotech), or HBD2 (Alpha Diagnostic). After incubation with the secondary antibodies, samples were labeled with protein A-10-nm gold, embedded, and observed with a JEOL 1400 electron microscope. Transmission EM of mouse liver tissues was performed as previously reported [24].

### siRNA, Anti-miRs, and miRNA Precursors

siRNAs to SNAP23, LL-37 and HBD2, and negative control oligos (Dharmacon) were used at a concentration of 10 nM and transfected with Lipofectamine RNAimax according to the manufacturer's protocol (Invitrogen). Anti-miRs and miRNA precursors (Ambion) were used to manipulate miRNA function in cells, as previously reported [27], [28], [41].

### Luciferase Reporter Constructs and Luciferase Assay

Complementary 39 bp for human and 33 bp for mouse DNA oligonucleotides containing the putative *let-7* family miRNAs target site within the 3′UTR of human SNAP23 (Human-Sense: 5′-ctag ACATGAATTCAGATTTACCTCAATGCTAAGAATTA-3′; Human-antisense:5′-agct TAATTCTTAGCATTGAGGTAAATCTGAATTCATGT-3′; Mouse-Sense: 5′-ctag CATGTGAATTCAGATTTACCTCAATACTA-3′; Mouse-antisense:5′-agct TAGTATTGAGGTAAATCTGAATTCACATG-3′) were cloned into the multiple cloning site of the pMIR-REPORT Luciferase vector (Ambion). Another pMIR-REPORT Luciferase construct containing mutant 3′UTR (CCTC to GGAG for human; ACCT to TGGA for mouse) was also generated as a control. Transfection and assessment of luciferase activity were performed as previously reported [27], [28], [41].

### Accession Numbers

The mRNA sequence data for genes described in this study can be found in the NCBI under the following accession numbers: *Homo sapiens* CD63 (NM_001257389), *Mus musculus* CD63 (NM_001042580); *Homo sapiens* TLR4 (NM_001257389), *Mus musculus* TLR4 (NM_025817); *Homo sapiens* Rab27b (NM_004163), *Mus musculus* Rab27b (NM_030554); *Homo sapiens* MyD88 (NM_001172566), *Mus musculus* MyD88 (NM_010851); *Homo sapiens* IKK2 (NM_001190720), *Mus musculus* IKK2 (NM_001159774); *Homo sapiens* Rab11 (NM_014904), *Mus musculus* Rab11 (NM_017382); *Homo sapiens* SNAP23 (NM_003825), *Mus musculus* SNAP23 (NM_001177792); *Homo sapiens let-7i* (NR_029661), *Mus musculus let-7i* (NR_029527); *Homo sapiens* miR-98 (NR_029513), *Mus musculus* miR-98 (NR_029753); *Homo sapiens* HBD2 (NM_004942); *Homo sapiens* LL-37 (NM_004345), *Mus musculus* LL-37 (NM_009921).

## Supporting Information

Figure S1 **Nanosight Tracking Analysis (NTA), Western blot for CD63 and MHC proteins, and release of apical exosomes from monolayers of different epithelial cell types following** ***C. parvum*** **infection or LPS stimulation.** (A) Size and particle distribution plots of isolated apical exosomes from the non-infected and *C. parvum*-infected (12 h) H69 monolayers by NTA. Both plots show a peak size around 100 nm for these isolated exosomes. Of note, the concentration scores for exosomes from non-infected and infected monolayers are different. Insert images are the representative screenshots of isolated exosomes by NTA. Image size recorded by video is approximately 100 nm. (B) Increased exosome release to the apical region following *C. parvum* infection was also confirmed by Western blot for CD63. (C) Increased amounts of MHC I and MHC II were detected in both the apical and basolateral exosomes released from H69 monolayers following infection. (D) Release of apical exosomes from monolayers of different epithelial cell types following *C. parvum* infection or LPS stimulation. Cells were grown to form monolayers on the Percoll inserts and then exposed to *C. parvum* infection for 24 h or LPS stimulation for 12 h. Exosomes were isolated from the apical supernatants and assessed by Western blot for CD63 (upper panel) and NTA (lower panel). *, p<0.05 ANOVA versus the non-infected controls.
(TIF)

Figure S2 **Association of SNAP23 with MVBs in H69 cells overexpressing SNAP23.** H69 cells were transfected with the SNAP23 plasmid and then exposed to *C. parvum* infection for 12 h. Cells were stained with CD63 antibody. Accumulation of SNAP23 around CD-63-positive multi-vesicular bodies was obvious as assessed by confocal microscopy. Scale bars = 5 µm.
(TIF)

Figure S3 **Expression of SNAP23 and phosphorylation of SNAP23 in 603B cells following** ***C. parvum*** **infection, and epithelial expression of miR-98 and anti-** ***C. parvum*** **activity of isolated exosomes from H69 monolayers after LPS stimulation.** Cells were grown to form monolayers on the Percoll inserts and then exposed to *C. parvum* infection or LPS stimulation. SNAP23 expression and its phosphorylation were assessed by Western blot and IP analysis (A and B). Expression of miR-98 was quantified by real-time PCR (C), and viability of *C. parvum* sporozoites after incubation with isolated exosomes was measured by epifluorescence microscopy (D). *, p<0.05 ANOVA versus the medium control.
(TIF)

Figure S4 **Anti-** ***C. parvum*** **activity of isolated exosomes from IEC4.1 monolayers following** ***C. parvum*** **infection.** Cells were grown to form monolayers on the Percoll inserts and then exposed *C. parvum* infection for 24 h. Released apical exosomes were collected and purified. These exosomes were then incubated with freshly excysted *C. parvum* sporozoites for 2 h, and after extensive washing, the sporozoites were added to IEC4.1 cells for infection. Parasite infection burden was measured by real-time PCR. *, p<0.05 ANOVA versus the medium control.
(TIF)

Figure S5 **Viability of** ***E. coli*** **K12 strain after incubation with isolated apical exosomes from** ***C. parvum*** **-infected H69 monolayers.** K12, a laboratory strain of *E. coli* susceptible to all antimicrobial drug classes, was incubated with apical exosomes isolated from non-infected or *C. parvum*-infected H69 monolayers. OD_600_ measurements were taken every 60 min for a total of 5 h. Recombinant human LL-37 was used as the positive control and phosphate buffered saline was used as a negative control. These viability measurements were substantiated by performing plate counts collected at each time point.
(TIF)

Text S1 **Details of Western blot, real-time PCR, Northern blot, ELISAs, immunoprecipitation (IP),** ***C. parvum*** **viability assay,** ***E. coli*** **viability experiments, and statistical analysis of data.**
(DOC)
