# Supplementary figures and images for: Release of Luminal Exosomes Contributes to TLR4-Mediated Epithelial Antimicrobial Defense
Source: PLoS Pathog. 2013 Apr 4;9(4):e1003261. doi: 10.1371/journal.ppat.1003261 (PMC3617097; doi:10.1371/journal.ppat.1003261)

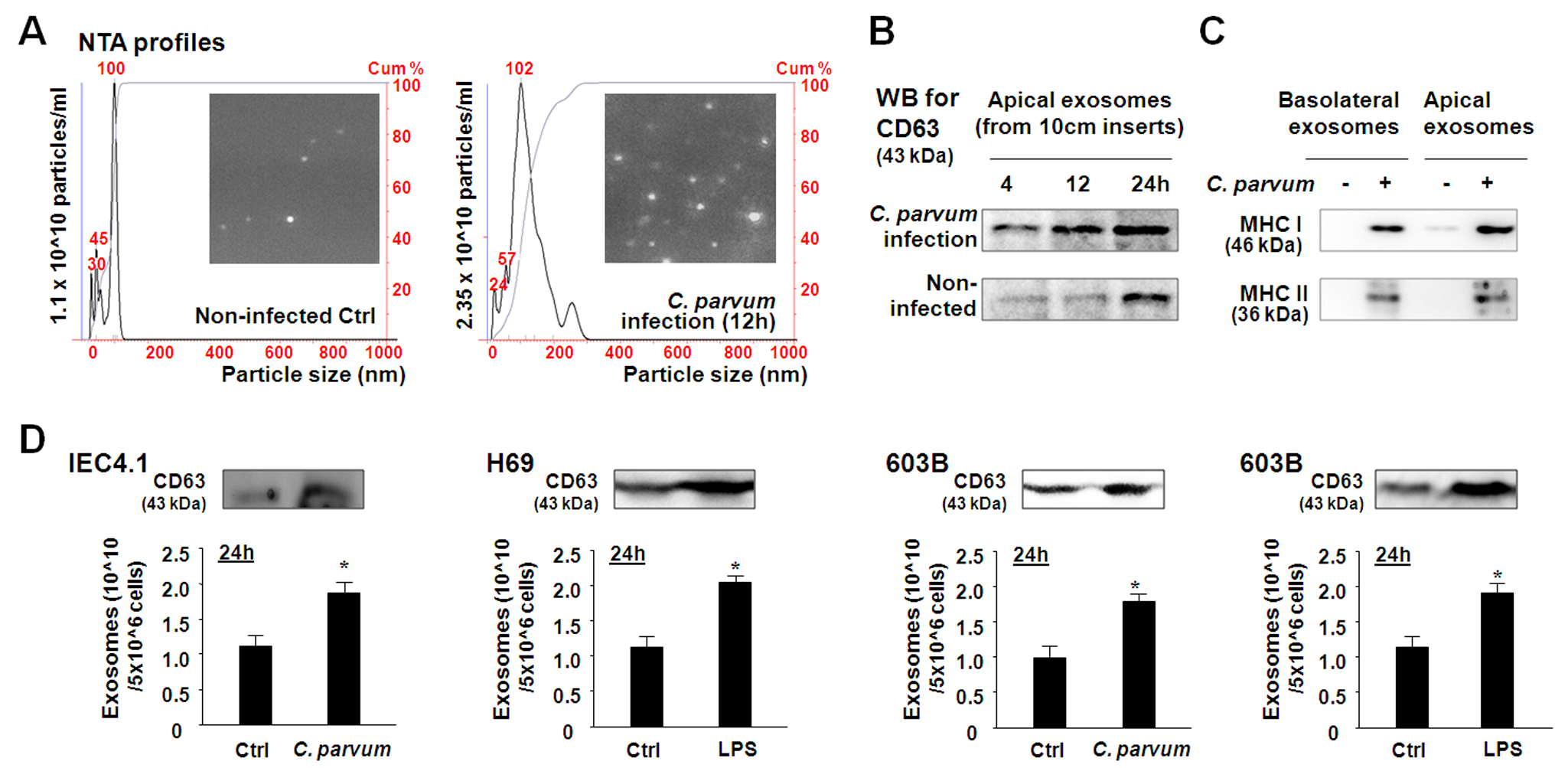

Supplement: Figure S1 — Nanosight Tracking Analysis (NTA), Western blot for CD63 and MHC proteins, and release of apical exosomes from monolayers of different epithelial cell types following C. parvum infection or LPS stimulation. (A) Size and particle distribution plots of isolated apical exosomes from the non-infected and C. parvum-infected (12 h) H69 monolayers by NTA. Both plots show a peak size around 100 nm for these isolated exosomes. Of note, the concentration scores for exosomes from non-infected and infected monolayers are different. Insert images are the representative screenshots of isolated exosomes by NTA. Image size recorded by video is approximately 100 nm. (B) Increased exosome release to the apical region following C. parvum infection was also confirmed by Western blot for CD63. (C) Increased amounts of MHC I and MHC II were detected in both the apical and basolateral exosomes released from H69 monolayers following infection. (D) Release of apical exosomes from monolayers of different epithelial cell types following C. parvum infection or LPS stimulation. Cells were grown to form monolayers on the Percoll inserts and then exposed to C. parvum infection for 24 h or LPS stimulation for 12 h. Exosomes were isolated from the apical supernatants and assessed by Western blot for CD63 (upper panel) and NTA (lower panel). *, p<0.05 ANOVA versus the non-infected controls. (TIF) [file ppat.1003261.s001.tif]

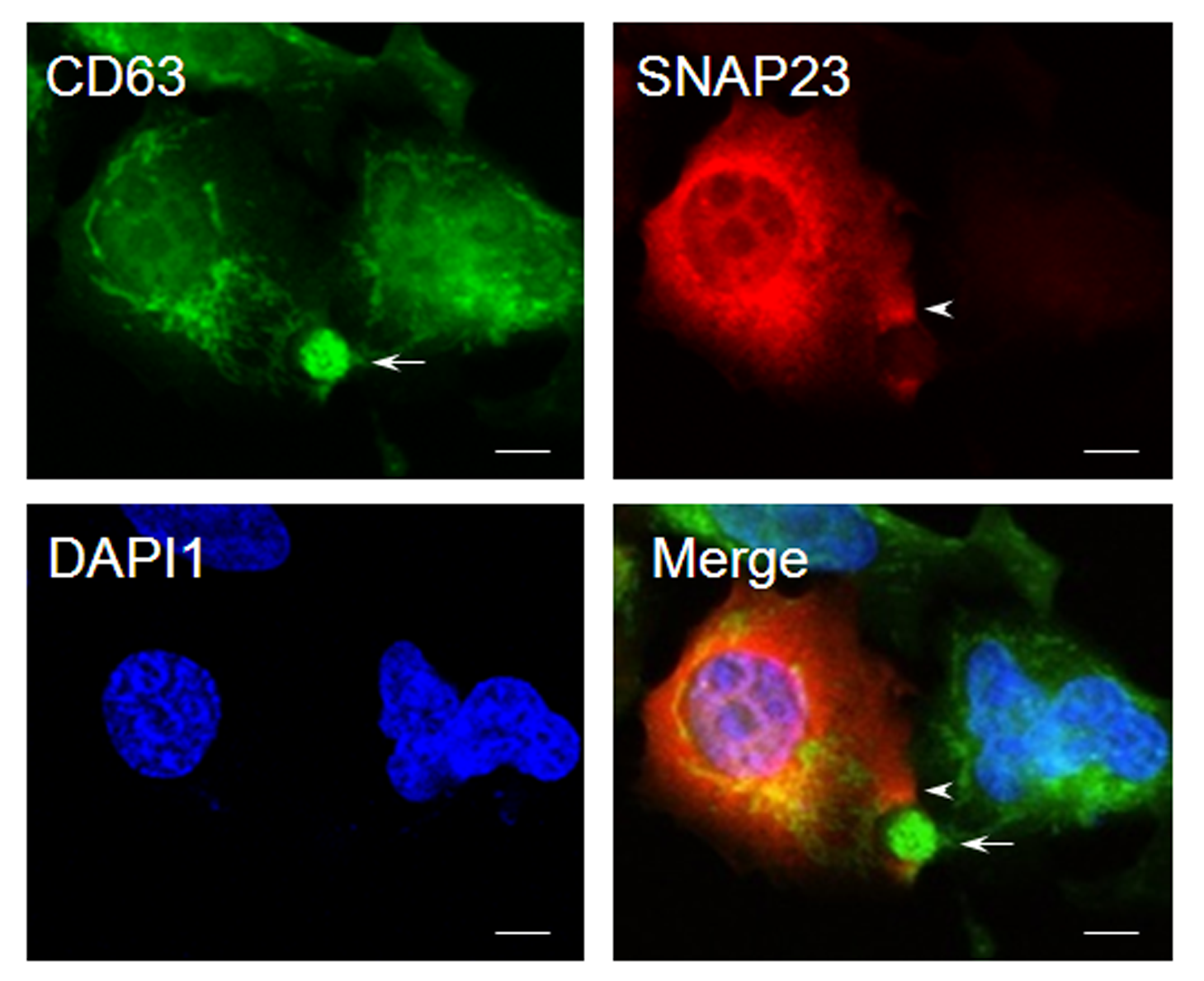

Supplement: Figure S2 — Association of SNAP23 with MVBs in H69 cells overexpressing SNAP23. H69 cells were transfected with the SNAP23 plasmid and then exposed to C. parvum infection for 12 h. Cells were stained with CD63 antibody. Accumulation of SNAP23 around CD-63-positive multi-vesicular bodies was obvious as assessed by confocal microscopy. Scale bars = 5 µm. (TIF) [file ppat.1003261.s002.tif]

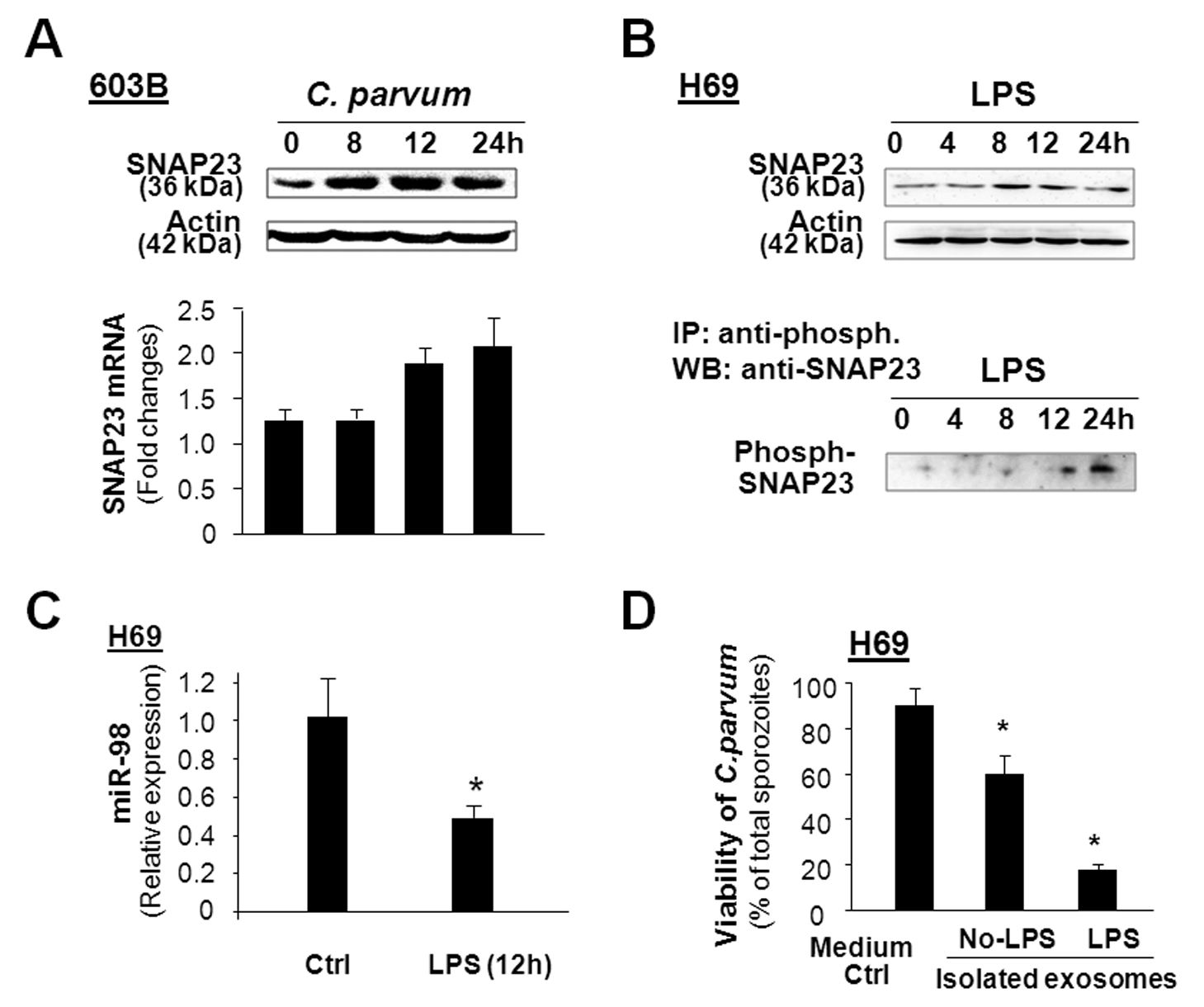

Supplement: Figure S3 — Expression of SNAP23 and phosphorylation of SNAP23 in 603B cells following C. parvum infection, and epithelial expression of miR-98 and anti- C. parvum activity of isolated exosomes from H69 monolayers after LPS stimulation. Cells were grown to form monolayers on the Percoll inserts and then exposed to C. parvum infection or LPS stimulation. SNAP23 expression and its phosphorylation were assessed by Western blot and IP analysis (A and B). Expression of miR-98 was quantified by real-time PCR (C), and viability of C. parvum sporozoites after incubation with isolated exosomes was measured by epifluorescence microscopy (D). *, p<0.05 ANOVA versus the medium control. (TIF) [file ppat.1003261.s003.tif]

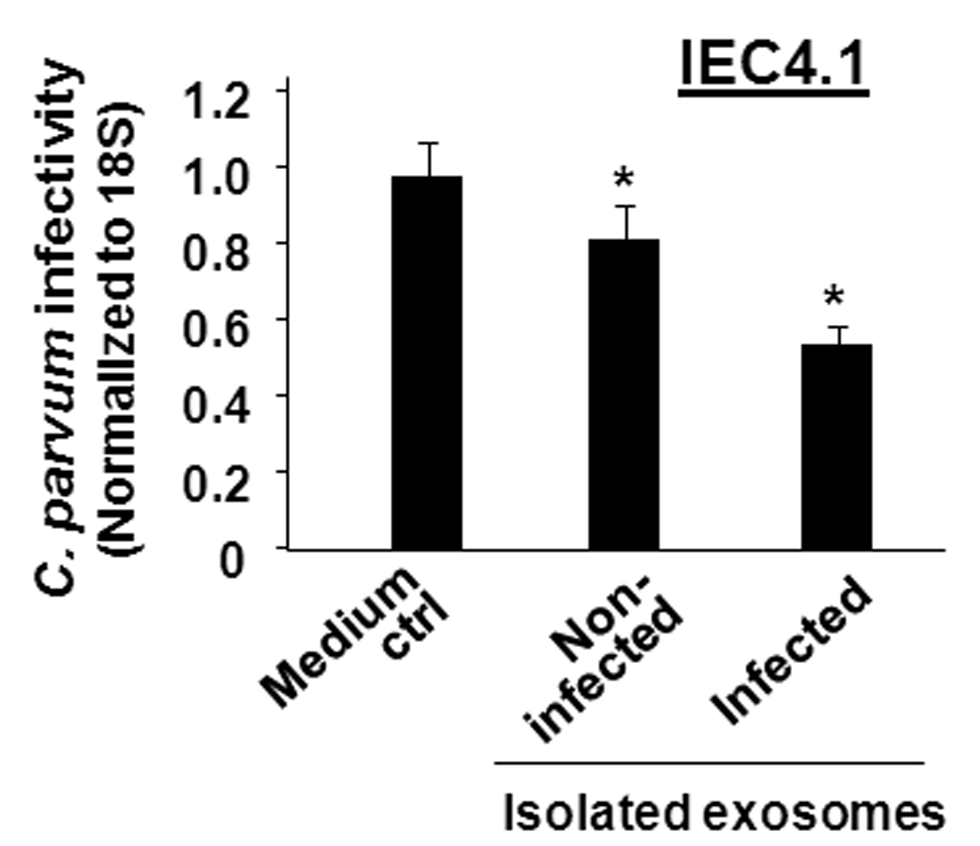

Supplement: Figure S4 — Anti- C. parvum activity of isolated exosomes from IEC4.1 monolayers following C. parvum infection. Cells were grown to form monolayers on the Percoll inserts and then exposed C. parvum infection for 24 h. Released apical exosomes were collected and purified. These exosomes were then incubated with freshly excysted C. parvum sporozoites for 2 h, and after extensive washing, the sporozoites were added to IEC4.1 cells for infection. Parasite infection burden was measured by real-time PCR. *, p<0.05 ANOVA versus the medium control. (TIF) [file ppat.1003261.s004.tif]

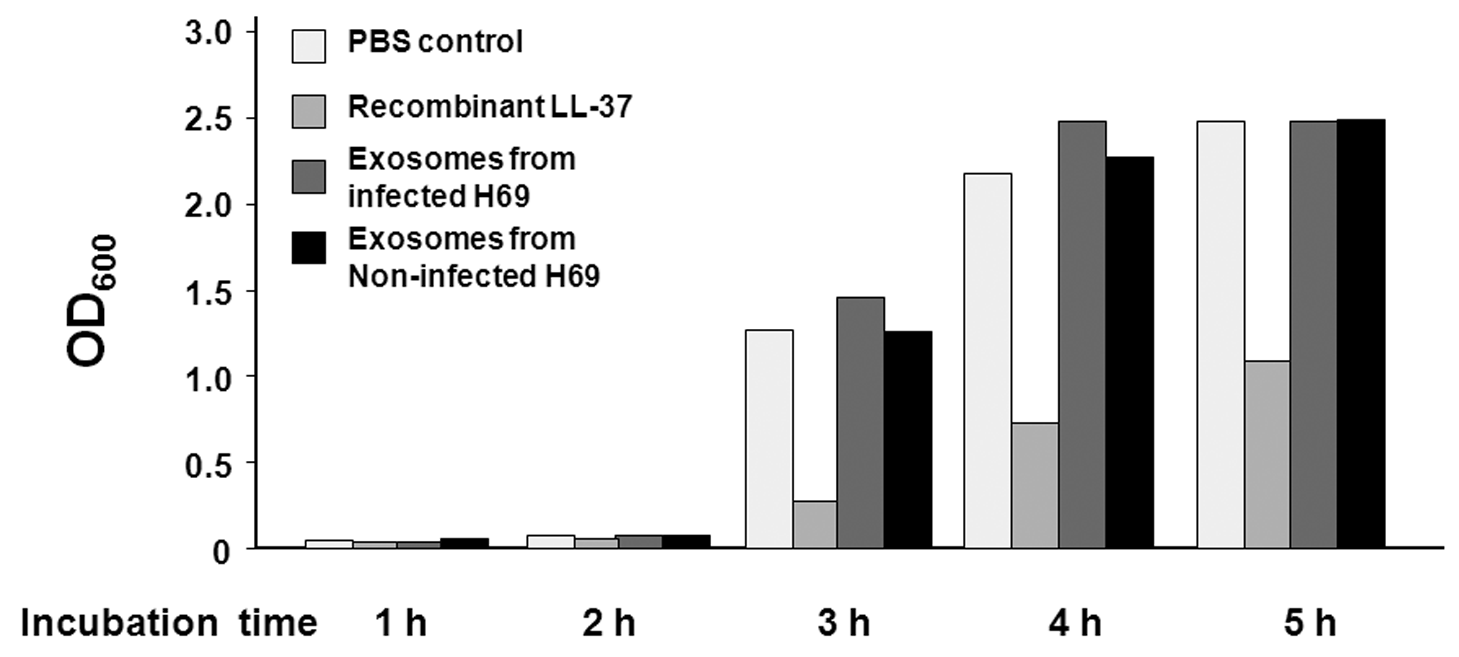

Supplement: Figure S5 — Viability of E. coli K12 strain after incubation with isolated apical exosomes from C. parvum -infected H69 monolayers. K12, a laboratory strain of E. coli susceptible to all antimicrobial drug classes, was incubated with apical exosomes isolated from non-infected or C. parvum-infected H69 monolayers. OD600 measurements were taken every 60 min for a total of 5 h. Recombinant human LL-37 was used as the positive control and phosphate buffered saline was used as a negative control. These viability measurements were substantiated by performing plate counts collected at each time point. (TIF) [file ppat.1003261.s005.tif]
