# Supplementary material for: Release of Luminal Exosomes Contributes to TLR4-Mediated Epithelial Antimicrobial Defense
Source: PLoS Pathog. 2013 Apr 4;9(4):e1003261. doi: 10.1371/journal.ppat.1003261 (PMC3617097; doi:10.1371/journal.ppat.1003261)
Supplement: Text S1 — Details of Western blot, real-time PCR, Northern blot, ELISAs, immunoprecipitation (IP), C. parvum viability assay, E. coli viability experiments, and statistical analysis of data. (DOC) [file ppat.1003261.s006.doc]

**Text S1 Details of Western blot, real-time PCR, Northern blot, ELISAs, immunoprecipitation (IP), *C. parvum* viability assay, *E. coli* viability experiments, and statistical analysis of data.**

**Western Blot.** Whole cell lysates were obtained from H69 cells with M-PER Mammalian Protein Extraction Reagent (Pierce) plus several protease inhibitors (1 mM PMSF; 10 μg/ml leupeptin, 2 μg/ml pepstatin). Cell lysates were then loaded at each line (a total of 40 μg lysate proteins) in 4-12% SDS page gel to separate proteins, and then were transferred to nitrocellulos membrane. Antibodies to SNAP23 (Santa Cruz), CD63 (Santa Cruz), MHC I (Santa Cruz), MHC II (Abcam), and actin (Sigma-Aldrich) were used. Densitometric levels of signals were quantified and expressed as their ratio to actin.

**Real-time PCR.** For real-time PCR analysis of mature miRNAs, total RNAs were extracted using the mirVana miRNA Isolation kit (Ambion). An amount of 0.05 μg total RNA was reverse-transcribed using the Taqman MicroRNA Reverse Transcription Kit (Applied Biosystems). Comparative real-time PCR was performed in triplicate using the Taqman Universal PCR Master Mix (Applied Biosystems) on the Applied Biosystems 7500 FAST real-time PCR System. Mature miRNA-specific primers and probes were obtained from Applied Biosystems. Normalization was performed using RNU6B primers and probes. Relative expression was calculated using the comparative CT method (1, 2).

**Northern Blot.** Total RNAs harvested as above were run on a 15% Tris/Borate/EDTA (90 mM Tris/64.6 mM boric acid/2.5 mM EDTA, pH 8.3) urea gel (Invitrogen) and transferred to a Nytran nylon transfer membrane (Ambion). An LNA DIG-probe of miR-98/*let-7* (Exiqon) was hybridized using UltraHyb reagents (Ambion) according to the manufacturer’s instructions, with blotted snRNA RNU6B as a control (3).

### **ELISAs.** Concentrations of HBD2 and LL-37 in exosomes were measured with ELISA kits from Alpha Diagnostic (San Antonio) and **Hycult Biotech** (**Uden**, **Netherlands**), respectively. Exosome protein lysates were stored at -20°C until use for ELISA, according to the manufacturer's instructions.

**Immunoprecipitation (IP).** Cells were lysed with the lysis buffer (20 mM Tris-HCl, pH 8.0, 150 mM NaCl, 1% NP-40, 20 μM MG132, 1 mM PMSF, 10 μg/ml leupeptin, and 2 μg/ml pepstatin). A total of 100 μg of lysate protein was incubated with an anti-phosphorylation antibody (Santa Cruz) at 4°C overnight to immunoprecipitate phosphorylated proteins. Immune complexes were collected by direct binding to protein A-Sepharose. The immunoprecipitates were then blotted with an anti-SNAP23 antibody (Invitrogen). For detection of SNAP23-IKK2 interaction, the anti-SNAP23 was used for immunoprecipitation and anti-IKK2 (Abcm) was used for blotting.

***C. parvum* Viability Assay.** Dye permeability assays using 4′6-diaminidino-2-phenylindole (DAPI) (Sigma) and propidium iodide (PI) (Sigma) were performed as previously described (4, 5). Briefly, sporozoites were pretreated with exosomes at room temperature for 2h, then these sporozoites were incubated with 10 μl of DAPI working solution (2 mg/ml in absolute methanol) and 10 μl of PI working solution (1 mg/ml in 0.1 M PBS, pH 7.2) at 37°C for 2h. Then sporozoites were washed twice in HBSS before being viewed by epifluorescence microscopy. Proportions of ruptured (‘ghost’), PI-positive (PI+, dead), and DAPI-positive PI-negative (DAPI+/PI−, viable at assay), DAPI-negative PI-negative (DAPI−/PI−, viable after further trigger) sporozoites were quantified by enumerating more than 100 sporozoites in each sample. Only the percentage of DAPI+/PI− sporozoites was considered as viable.

***E. coli* Viability Experiments.** Viability experiments were performed using the laboratory strain *Escherichia coli* K12, which is susceptible to all antimicrobial drug classes. Customized side-arm flasks, in which the end of the flask has been fused to a pyrex cuvette to ensure the consistency of volume, were used for the optical density (OD_600_) measurements. Each experiment included four flasks with 10 μl of overnight liquid culture inoculated into 10 ml of Mueller Hinton Broth (pH 5.5): culture with 500 μl phosphate buffered saline (mock control), culture within the presence of recombinant human LL-37 (positive control), culture with 500 μl of exosomes isolated from H69 monolayers after *C. parvum* infection, and culture with 500 μl of exosomes isolated from uninfected H69 monolayers. Each 10 ml of culture was grown to an OD_600_ of 0.035, which represents early-log phase growth. OD_600_ measurements were taken every 60 min for a total of 5h. The antimicrobial effect of these exosomes was assessed by changes observed in the OD_600_ of the culture over time, as in the previous studies (6,7). These viability measurements were substantiated by performing plate counts collected at each time point, as previously reported (6,7).

**Statistical analysis of data.** Groups of data were compared using the ANOVA test. p<0.05 was considered to represent statistical significance.
